# Active Commuting to School among Spanish Preschool Children: A Temporal Change Study between 2013 and 2017 †

**DOI:** 10.3390/children11010003

**Published:** 2023-12-20

**Authors:** Romina Gisele Saucedo-Araujo, Patricia Gálvez-Fernández, Cristina Cadenas-Sanchez, Mairena Sánchez-López, Pau Avellaneda, Josep M. Suelves, Francisco Javier Huertas-Delgado, Palma Chillón, Manuel Herrador-Colmenero

**Affiliations:** 1 Department of Didactics of Musical, Plastic and Corporal Expression, Faculty of Education and Sport Sciences, University of Granada, 52005 Melilla, Spain; rgs@ugr.es; 2 Department of Physical Education and Sports, Faculty of Sport Sciences, Sport and Health University Research Institute (iMUDS), University of Granada, 18011 Granada, Spain; pgalvez@ugr.es (P.G.-F.); cadenas@ugr.es (C.C.-S.); pchillon@ugr.es (P.C.); 3 Institute for Innovation & Sustainable Development in Food Chain (IS-FOOD), Department of Health Sciences, Public University of Navarre, IdiSNA, Navarra Institute for Health Research, 31006 Pamplona, Spain; 4 Health and Social Research Centre, Universidad de Castilla-La Mancha, 16071 Cuenca, Spain; mairena.sanchez@uclm.es; 5 School of Education, Universidad de Castilla-La Mancha, 13071 Ciudad Real, Spain; 6 Departament de Geografia, Universitat Autònoma de Barcelona, 08193 Barcelona, Spain; pau.avellaneda@uab.cat; 7 Health Department, Public Health Agency of Catalonia, Government of Catalonia, 08005 Barcelona, Spain; josepmaria.suelves@gencat.cat; 8 eHealth Center Behavior Design Laboratory, Universitat Oberta de Catalunya—UOC, Rambla del Poblenou, 08018 Barcelona, Spain; 9 La Inmaculada Teacher Training Centre, University of Granada, 18011 Granada, Spain

**Keywords:** active transport, walking, trends, school, kindergarten

## Abstract

Background: Active commuting to school may increase the total daily physical activity and achieve health benefits among preschool children. Rates of active commuting to school among Spanish children and adolescents have been widely analysed, while the rates of active commuting to school among Spanish preschool children are unknown. Aim: The main objective of this study was to examine the changes in the rates of active commuting to school in a sample of Spanish preschool children between 3 and 6 years old from 2013 to 2017. Methods: Data were found from five studies carried out across Spain. The study sample comprised 4787 preschool children (4.59 ± 0.77 years old; 51% males). The overall changes in active commuting to school were assessed using multilevel logistic regression analysis. Results: The rates of active commuting to school in Spanish preschool children are around 52%, and the active commuting to school rates have stayed stable throughout the period assessed (odds ratio from 0.40 to 0.58, all *p* > 0.05). Conclusion: In preschool children, the present study obtained a favourable result on active commuting to school, showing a pattern stability in the examined period similar to other ages. It will be of great importance to promote this behaviour to obtain high levels of active commuting to school.

## 1. Introduction

The scientific evidence reports that daily physical activity is positively associated with multiple healthy effects in preschool children (i.e., children under 6 years old), such as physical, psychological, social, and cognitive benefits [1,2,3,4,5,6]. Specifically, regular physical activity in preschool children prevents obesity [7], contributes to the development of motor skills, raises psychosocial health, and improves cardiometabolic health indicators [8], thus contributing to the correct development of young children [5]. Furthermore, early childhood growth is a strong determinant of long-term health outcomes in adulthood [9]. Physical activity guidelines for preschool children have been recently developed in numerous countries [10,11,12,13]. The recent recommendations proposed by the World Health Organization [14] indicate that preschool children, schoolchildren, and adolescents may achieve physical activity benefits by reaching an average of at least 60 minutes per day of moderate-to-vigorous physical activity throughout aerobic activities. Thus, the guidelines provide a tool to bring all behaviours along the movement continuum [15]. Moreover, a systematic review in South America pointed to high exposure to sedentary behaviour in preschool children and recommended that they should be physically active for at least three hours each day, with activity spread across the day. In addition, preschool children need to be physically active between 1 and 3 h each day, with energetic play such as running, jumping, or walking [16]. There is no clear guideline like there is for older ages, so different countries are concerned about finding clear recommendations for this age group. As a result, guidelines have been developed for the preschool age in some countries [10,11,13], but it is unclear how much physical activity is considered sufficient for preschool children [17].

Around 84% of the children (2–9 years old) in the IDEFICS Study (European Identification and Prevention of Dietary- and Lifestyle-Induced Health Effects in Children and Infants) [18] did not meet the physical activity guideline recommendations. Instead, Hnatiuk et al. [19] noted that preschool children (2–5 years old) spend only 2–41% of their day in moderate-to vigorous physical activity intensity and 4–33% in light physical activity intensity. In the systematic reviews developed by Sallis et al. [20] and Pate et al. [21] about children’s physical activity, they found that boys were more physically active than girls. It is difficult to determine the “true” prevalence of physical activity and sedentary time in preschool children [19]. However, there is a notable deficiency in the amount of physical activity recommendation guidelines for the health of preschool children [17,22].

Chaput et al. [23] had observed that more time spent in sedentary behaviour, to be more specific, recreational screen time, is associated with poorer health outcomes in both children and adolescents. For example, longer durations of television viewing are associated with poorer physical fitness. The longer they engage in this behaviour, the more they may be associated with poorer mental health and negative prosocial behaviour. The many benefits of regular practice of physical activity on the health of the general public and also on children and youth are widely known. The early childhood education stage (<6 years old) is a relevant stage for learning habits through routines, thus helping as an opportunity to establish healthy lifestyles based on physical activity behaviours [24]. In Europe, 7.9% of preschool children are overweight or obese [25]. In response to this situation of physical inactivity among children and young people, many governmental organizations have put forward numerous physical activity proposals. Specifically, the International Society for Physical Activity and Health (ISPAH) [26] in 2020 proposed eight investment strategies to ensure people’s health through the practice of physical activity. Among them were, firstly, the promotion of interventions in the school environment and, secondly, strategies to promote transport or active travel.

These proposals are in line with what Sallis [27] called the socio-ecological model with four dimensions for active living. Transport is one of the dimensions, which, at the individual level, is also one of the opportunities to be physically active. Therefore, an opportunity for young people to practice physical activity through active transport to and from one of their natural and habitual environments, like school, is what is known as active commuting to and from school. One of the current definitions carried out by Ruiz-Hermosa et al. [28] is as follows: “The use of active modes of transport, such as walking, cycling, skateboarding or other non-motorised modes of transport that involve energy expenditure during the journey to and from school”.

A new and promising strategy called Active Commuting to School began to be introduced in society in the early 2000s to increase daily physical activity levels among children and adolescents (i.e., 6- to 18-year-olds) [29,30]. Research has demonstrated that active commuting to school could provide substantial health benefits. In the scientific literature, active commuting to school has already been studied as a physical activity habit that is positively associated with several variables, such as physical fitness [30,31] and individual happiness [32] as well as psychosocial [33] and environmental variables [34]. Therefore, as promoted by Chillón et al. [35,36] since 2010 and other authors [37], active commuting to school is an opportunity to increase the health of young people.

Over the last two decades, several studies have concluded that active commuting to school can contribute to total physical activity. For example, Lee et al. [38] showed a positive association between active commuting and overall physical activity levels in children. Similar results were shown in a systematic review carried out by Faulkner et al. [39]; they concluded that active school commuters tend to be more physically active overall than passive commuters. Furthermore, active commuting to school enhances children and adolescents’ autonomy and independence (i.e., better psychological health and self-efficacy) [40].

The European IDEFICS study [28] showed that the prevalence of active commuting to school was 46% in Spanish children (an average age of 5.3 years old). According to Pabayo et al. [41], the prevalence of students who use active commuting to school increases from 6 years old and reaches a maximum at the age of ~10 years old, and then it decreases throughout adolescence. In contrast with the wide evidence on the rates of active commuting to school behaviour among children and adolescents, fewer studies have focused on preschool children. The active commuting to school rates among preschool children (5 years old) were 37% in New Zealand [42], while in Brazil, 28% of preschool children commuted actively from home to school [43]. However, in a recent study [44], approximately 70% of Colombian children and adolescents between 3 and 17 years reported engaging in active commuting to/from school over the last week. In Europe, the IDEFICS study [45] analysed active commuting to school in preschool children (2 to 6 years old) in eight countries between September 2007 and June 2008. Four countries (Italy, Estonia, Belgium, and Cyprus) reported a low percentage of active commuting to school, between 6% and 20%. On the other hand, higher percentages, between 25% and 35%, were reported in Germany, Sweden, and Hungary, and Spain had the highest rate of active commuting to school (54%). Analysing the recent evidence in Spain, the MOVI-KIDS study found in 2013 that 46% of preschool children walked to school [28], and Terrón-Perez et al. [46] found that almost 70% of Spanish preschool children had active commuting to school behaviour between 2015 and 2016. Evidence about the active commuting to school trends in preschool children around the world is scarce. Recently, the MoMo Study [47], developed in Germany, analysed the active commuting to school trends from childhood to adolescence (from 4 to 17 years old) between 2003 and 2017. In both boys and girls aged 4–5 years old, the active commuting to school rate significantly decreased from 2003 to 2017 (boys: from 85% to 78%; girls: from 84% to 77%). However, there is additional evidence about the active commuting to school trends in children and adolescents. The Health Behaviour in School-Aged Children [48], developed in the Czech Republic, Norway, Scotland, and Wales, analysed data from 88,212 students (11, 13, and 15 years old). This study shown stable patterns of active commuting to school from 2006 to 2018, in addition to a decrease in the Czech Republic between 2006 and 2010. In a recent trend study carried out in Spain [49], the rates of active commuting to school in Spanish children and adolescents did not change significantly during the 2010–2017 period, except for an occasional increase in the rate of active commuting to school in adolescents in 2012–2013. Hence, it is important to understand how active commuting to school behaviour has changed in the last years among Spanish preschool children in order to develop appropriate educational strategies and promotion programmes focused on the achievement of physical activity guidelines and the adoption of healthy lifestyles in this population [50]. Thus, the aims of this study were to describe the rates of active commuting to school and to review the changes in the rates of active commuting to school between 2013 and 2017 among Spanish preschool children.

## 2. Materials and Methods

### 2.1. Study Design

The study explored cross-sectional data about Spanish preschool children’s modes of commuting to and/or from school from research centres and local/regional public institutions between 2013 and 2017. The current study is part of the PACO Study (Cycle and Walk to School Study) that aims to (a) describe the rates of active commuting to school among Spanish children and adolescents and (b) promote active commuting to school through interventions in the school context [51].

### 2.2. Procedure

The design and methodology of the current study have already been described in detail elsewhere [49]. Briefly, those research centers and local/regional public institutions (hereafter, studies) offered the following data: the data collection date (month and year) and school location, as well as the participants’ age, gender, mode of commuting to school, and socioeconomic status, if available. In addition, the research team for this study collected information on population density and income in the localities where the schools were located. The inclusion criteria for including these studies were as follows: (a) data were collected using a questionnaire completed by the children’s parents; (b) data were available at the participant level; (c) studies provided data collected until the end of the recruitment period (i.e., 2017); and (d) studies provided data on the data collection date and school location, as well as preschool children’s mode of commuting to school, age, and gender. Of the 34 initial studies that contributed data, 28 met the inclusion criteria.

The study followed the ethical considerations of Research in Sports Science and Exercise [52] according to the principles included in the Declaration of Helsinki [53]. The Medical Ethics Committee of University of Granada approved the PACO Study design, protocols, and informed consent procedure (case no. 162/CEIH/2016) on 6 June 2016.

### 2.3. Sample

**Figure 1:**
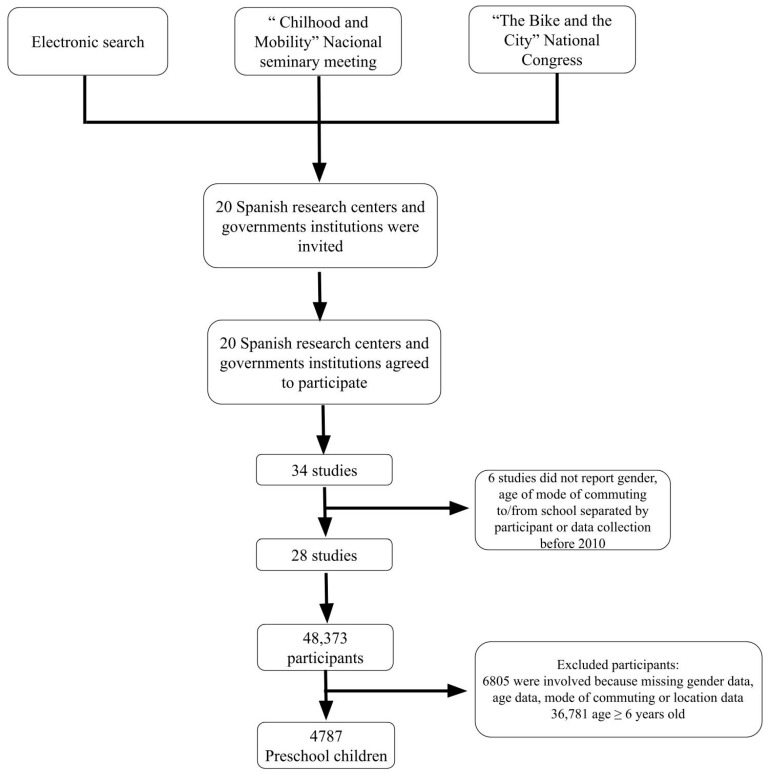
Flow chart of the study participants.

Data on 48,373 Spanish potential participants (preschool children (3.00 to 5.99 years old), children (6 to 11.99 years old), and adolescents (12 to 18 years old)) from 28 cross-sectional original projects carried out between 2010 and 2017 were collected. To be involved in the study, the inclusion criteria compulsory for participants were (a) to have completed information about gender and age, (b) to identify the school location, (c) to identify the mode of commuting and to be between 3.00 and 5.99 years old. Participants who did not meet some of the inclusion criteria were not included in the final sample. This study’s final sample included 4787 preschool children between 3.00 and 5.99 years old (males: 51.2%) from 27 different Spanish localities between 2013 and 2017 (Figure 1). Table S1: Description of the main characteristics of 5 studies in this study.

### 2.4. Measures

**Figure 2:**
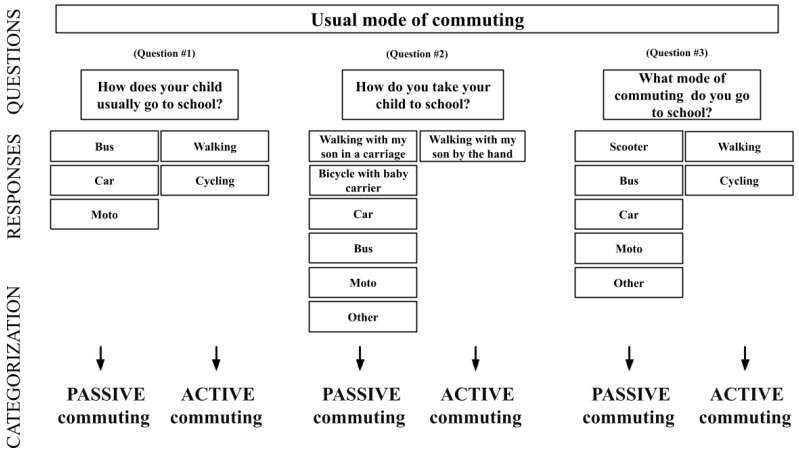
Questions to assess the mode of commuting.

The mode of commuting to and from school was evaluated using 3 different questions answered by parents through the studies (Figure 2). The questions used were as follows: (a) “How does your child usually go to school?” (b) “How do you take your child to school?” (c) “What mode of commuting do you use to go to school?” The current study only used the usual mode of commuting to school for analyses. Participants who identified walking (with the child by the hand), cycling, and/or using a nonmotorized scooter to travel to school were classified as active commuters. Participants who identified travelling to school by foot with the child in a carriage, bike with a baby carrier, school bus, public bus, train/metro, taxi, motorbike, and/or car were categorized as passive commuters. Participants using multiple modes as part of their school journey (i.e., using 2 or more different modes of commuting for the same trip) were excluded in the study [36]. This categorization was based on the characteristics of the mode and frequency of commuting to and from school questionnaire, which is a valid and reliable instrument to assess active commuting to school behavior [54,55].

Several sociodemographic characteristics were considered. Participants’ gender and age were recorded as individual data. Characteristics of the school location (i.e., population density and income) were estimated. Population density (number of inhabitants per locality area in km^2^) was obtained from the Ministry of Finance and Public Administration of Spain using the available information closest to the data collection year in each locality. Furthermore, the localities’ income of the data collection year was found from the Spanish Public Tax Agency (https://www.agenciatributaria.es; accessed on 12 December 2023). Each variable (population density and income) was categorized into a dichotomous variable (low vs. high) using the median.

In the present study, the survey year was described as the specific year when data were collected. The survey year was collected in each individual study. The years of the studies finally included were from 2013 to 2017.

### 2.5. Statistical Analysis

Descriptive statistics of the participants were identified for gender, age, population density, localities’ income, and mode of commuting; means and standard deviations were calculated for continuous variables; and percentages were reported for categorical variables. Additionally, active commuting to school by survey year was analysed using chi-squared analyses for the whole sample, as well as for boys and girls, respectively. As the sampling frame of participants was based on localities (i.e., participants nested in localities), relationships between active commuting to school and the survey year were assessed using multilevel logistic regression, with 2 levels for analysis: participants (level 1) and localities (level 2). Multilevel models have been proposed as the most appropriate analysis methodology for dealing with hierarchical data [56]. The goodness of fit of the empty model was studied, displaying an intraclass correlation of 0.20, which indicates that 20% of the variance in active commuting to school was explained by between-localities differences. Socioeconomic variables did not have an effect on the different localities (*p* > 0.05), as observed through the Wald test. The variable of active commuting to school became part of the models as the dependent variable and the survey year as the independent variable. Age and gender were included as covariates. The potential interactions between age, gender, and localities’ income were analysed in the multilevel logistic regression analyses, and there were not significant differences (*p* > 0.05). Statistical analysis was performed using STATA v.13 (Stata Corp: 110th edition College Station, TX, USA: Stata Corp LP; 2009, n.d.), and the statistical significance level was set at *p* < 0.05.

## 3. Results

Descriptive characteristics of the participants and localities are displayed in Table 1. Boys were 52% of the total sample, with a sample mean age of 4.59 years old. Population density was 2913.33 (hab/km^2^), and the localities’ income median was 27,881 euros.

**Figure 3:**
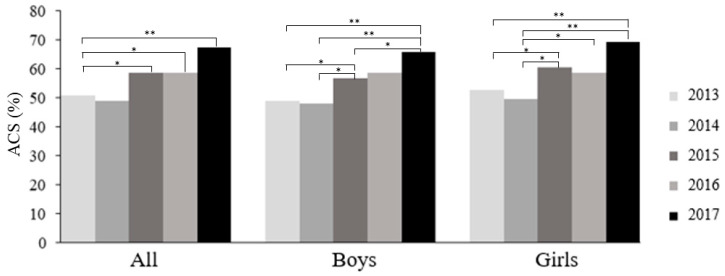
Active commuting to school in Spanish preschool children by years in the period 2013–2017 and by gender. * *p* < 0.05, ** *p* < 0.01, no significant differences were found in the same year by gender.

The active commuting to school rates in Spanish preschool children by survey years in the period 2013–2017, controlled for age, are shown in Figure 3. Overall, around 52% of the preschool children actively commuted to school between 2013 and 2017. The active commuting to school rate was around 51% in 2013 and 2014, around 58% in 2015 and 2016, and 67% in 2017. Active commuting to school differences by survey year were found for both boys and girls separately. In boys, a higher percentage of active commuting to school was found in 2015 vs. 2013 and 2014, as well as in 2017 vs. 2013, 2014, and 2015 (all, *p* < 0.05). Regarding girls, a higher percentage of active commuting to school was found in 2015 vs. 2013 and 2014, in 2016 vs. 2014, and in 2017 vs. 2013 and 2014 (all, *p* < 0.05). Moreover, active commuting to school differences by gender in the same survey year were studied, and no significant differences were found.

Associations between active commuting to school and survey years, adjusted for age and gender, are shown in Table 2. It was noted that there was no relationship of active commuting to school behaviour with the survey year during the period analysed (all, *p* > 0.05).

## 4. Discussion

The present study analyses the active commuting to school rates and changes among Spanish preschool children from 2013 to 2017 using data from 27 different localities. A total of 52% of the participants commuted actively to school (from 48.8% to 67.4%) during the period considered, and differences in the active commuting to school rate were found by survey year. However, no associations between the active commuting to school rates and the survey year were found for the Spanish preschool children.

The results of the present study indicate similar rates of active commuting to school, contrasted with previous studies conducted in Spain, such as the MOVI-KIDS study, which determined that 46% of preschool children walked to school [28], or the IDEFICS study, where almost 50% of preschool children actively commuted [57]. Instead, Terrón-Perez et al. [46] determined a higher rate of active commuting to school, in which 70% of preschool children commuted actively to school. There is little in the scientific literature on modes of active commute to and from school in preschool children. A study on children aged 2–3 years old found that parents reported the mode of daily transport, with cars and prams predominating in young children [58]. In other countries, higher active commuting to school rates were found in Germany, where more than 70% of preschool children aged 4–5 years old commuted actively [47], or in the United Kingdom, where 70% of children aged 5–10 years old reported walking to and from school [59]. However, in New Zealand, a lower rate of active commuting to school (40%) was found among preschool children (5 years old) [42] compared with the current results.

A change in active commuting to school in Spanish preschool children did not appear between the 2013–2017 period studied. In a study with German children who were 4–5 years old, active commuting to school significantly decreased from 2003 to 2017 (from 84% to 77%) [47]. The current study displays promising results as a decreasing change was not seen, which might be a starting point to obtain an active commuting to school rate increase in the next upcoming years. The difference in the results found in this study could be due to the increase in the number of German preschool children who were driven by their parents in this period [47]. Other trend studies focused on preschool children have not been discovered. Comparing the current results with previous studies focused on children (aged 6 to 12 years old), there is a decrease in the rate of active commuting to school in the countries observed such as the United States [60], China [61], Australia [62], and Germany [63]. An increased distance from home to school appears to be a key contributor to this decline [64], and the distance could be influenced by various barriers [65]. It is unknown if this perceived barrier is similar in the families of preschool children. Among preschool children, the parents choose how they should go to school [66,67], but when they grow up, they can increase active commuting to school in various ways, such as by walking to school with friends, going somewhere with friends after school, or travelling to the park to play. When children grow older, their parents perceive a decrease in control and supervision [68] and an increase in autonomy [69].

Another reason could be the time period analysed in this study (i.e., between 2013 and 2017), which was dissimilar to previous studies, and it is connected to the recovery process of the economic crisis [70]. The stability in the active commuting to school rates detected in Spanish preschool children in recent years is consistent with the findings from Gálvez-Fernandez et al. [49] in Spanish children and adolescents. In Spain, the rate of active commuting to school continued to be stable from early ages up to the beginning of adolescence. The integration of preschool children and schoolchildren into the same school might be one cause for the similarity of findings in these age groups. Additionally, the establishment of active modes of commuting in preschool children could have a direct impact in the following ages. The stable trend in Spanish preschool children could also be due to the impact and influence of educational programmes on lifestyles [71]. In previous years from the time period analysed in the study, various proposals were applied to promote active commuting to school as a positive alternative to increase daily physical activity levels and other variables, from preschool children to adolescents, in Spain. Furthermore, interventions in this area increased in the near age group [30,72,73] and could contribute to the importance of commuting actively. Thus, advocacy among young people fosters greater social awareness of the importance of promoting physical activity and its benefits. The promotion programmes to increase physical activity could have an influence on the active commuting to school stability in this study; for example, “school path” and/or “pedibus” have been used among preschool children and adolescents in many cities in Barcelona since 2007 (https://www.uco.es/investigacion/proyectos/appedibus/otros-proyectos/proyectos-nacionales/caminos-escolares-de-cataluna/caminos-escolares-de-barcelona/camino-escolar-de-barcelona-ciudad/escuela-dolors-moserda-santapau-2/; accessed on 1 November 2023) and Valencia within a different municipality (https://laribera.san.gva.es/camino-escolar-tu-hijo-necesita-ir-al-cole-en-coche/; accessed on 10 the November 2023), as well as in San Sebastian or Madrid. A handbook on “safe school routes” has recently been developed with related objectives such as reducing the use of motorised vehicles for school travel and improving air quality and road safety around schools because of previous proposals carried out in Spain (https://www.miteco.gob.es/es/ceneam/recursos/materiales/manual-didactico-caminos-escolares-seguros.html; accessed on 27 the September 2023).

### Strengths, Limitations, and Perspectives of Future Research

Interpretations of the results of the present study must take into account several strengths and some limitations. The head strength of this study is that it is the first Spanish research analysing active commuting to school in preschool children covering a period of 5 years. Furthermore, the inclusion of a large sample of Spanish preschool children enabled this study to assess in detail the changes in active commuting to school using multilevel analyses. The main limitation was that it is not a nationally representative study but a geographically distributed one, although the number of participants varies from every year and region. Its cross-sectional design does not allow for the assumption of causal relationships. A longitudinal design would be more appropriate but requires more financial solvency, management, and support from social agents. Moreover, sampling procedures and original data from the various studies were not identical. In this respect, the mode of commuting to and/or from school was measured using different questions in the original studies, and the data collection also varies. In the current study, different methods of non-validated questionnaires have been unified. However, diversity of questions was categorized following the guidelines of the validated questionnaire [54,55]. Nevertheless, a systematic process was followed to merge the studies, and various questions were used to ensure accurate data and applicable analysis of these data. Another limitation is the inability to collect data about the commuting distance to control for the analyses; the main determinant of active commuting to school is a result of this.

Future research could analyse the changes within a time period using a representative sample of Spanish preschool children and using objective tools. According to the systematic review by Campos-Garzón et al. [74], in order to measure the route objectively, it is necessary to recognise the space-time in which the route takes place with the combination of a geolocation device (global positioning system). An accelerometer could also be incorporated to study the intensity with which preschool children travel to school to recognize more firmly how active commuting to school in these ages contributes to reaching the daily physical activity guidelines. The questionnaires are used due to the impossibility of using objective tools; it would be significant to use a valid and reliable questionnaire that evaluates the mode of commuting in order to compare between numerous schools. According to the systematic review [74], to reduce measurement errors and to be able to establish when the participant starts and ends their journey or to know the route between home-school-home, data from an accelerometer and a GPS could be combined if the budget is available. As another less expensive option, the use of more affordable activity wristbands could be considered in a school context. Future research should continue analysing active commuting to school change to understand the factors that influence this behaviour among preschool children (i.e., environmental characteristics, time, safety, or traffic). Recently, Lam et al. [75] indicated that future research should establish a standardised definition of active commuting to school, as well as analyse different forms of active modes among children’s groups in different areas (rural, regional, and developing). The school context represents an occasion to promote health-related behaviours, such as active commuting to school, from an early age [76]. Interdisciplinary work between teachers, parents, and communities, in order to be successful, is necessary for the promotion of active commuting. Current scientific literature confirms that effective physical activity programmes could lead to considerable savings for public health services [77].

## 5. Conclusions

The current research noticed that the rates of active commuting to school in Spanish preschool children were around 50% between 2013 and 2017, and these rates have remained stable. This study highlighted the significance of promoting active commuting to school from early stages to create healthy habits that ensure a healthy adulthood. Actions focused on a strong alliance between researchers and schools are required to reach high levels of active commuting to school.

## Figures and Tables

**Table 1:** Descriptive characteristics of the study participants and cities by survey year.

Preschool Children	All *n* = 4787	2013 *n* = 735	2014 *n* = 2867	2015 *n* = 634	2016 *n* = 198	2017 *n* = 353	*p*
Age ($\underline{X}$ ± SD)	4.59 ± 0.77	4.88 ± 0.33	4.53 ± 0.84	4.72 ± 0.62	4.16 ± 0.84	4.5 ± 0.81	<0.001
Gender							0.264
Boy [*n* (%)]	2498 (52.2)	403 (54.83)	1503 (52.42)	323 (50.95)	94 (47.47)	175 (49.58)	
Girl [*n* (%)]	2289 (47.8)	332 (45.17)	1364 (47.58)	311 (49.05)	104 (52.53)	178 (50.42)	
Population Density (median) (hab/km^2^)	2913.33	1048.367	2724.802	4320.324	11,505.59	3093.23	<0.001
Localities’ Income (median) (euros)	27,881	20,520	31,820	23,197	23,104	21,751	<0.001

$\underline{X}$, mean; SD, standard deviation.

**Table 2:** Associations of active commuting to school with survey years for preschool children adjusting by age and gender.

Preschool Children	*n* = 4787	OR	95% CI	*p*
Survey year				
2017	353	1	Reference	
2016	198	0.47	0.72–3.05	0.428
2015	634	0.58	0.92–3.63	0.559
2014	2867	0.53	0.83–3.36	0.498
2013	735	0.40	0.63–2.52	0.329
Age		1.10	1.02–1.20	0.019
Gender (Girl)		1.07	0.95–1.21	0.249

OR, Odds Ratio; CI, Confidence Interval.

## Data Availability

The data presented in this study are available on request from the corresponding author. The data are not publicly available due to privacy issues.

## Supplementary Materials

The following supporting information can be downloaded at https://www.mdpi.com/article/10.3390/children11010003/s1: Table S1: Description of the main characteristics of each study included.
