# Supplementary material for: Active Commuting to School among Spanish Preschool Children: A Temporal Change Study between 2013 and 2017
Source: Children (Basel). 2023 Dec 20;11(1):3. doi: 10.3390/children11010003 (PMC10813918; doi:10.3390/children11010003)
Supplement: Supplementary file 1 [file children-11-00003-s001.zip › children-2747880-supplementary.pdf]

Supplementary Material

Table S1. Description of the main characteristics of each study included.

Study name	Research centre (RC)/ Government institution (GB)	Collected data (year)	Localities	Preschool children (sample)
MOVIKIDS	University of Castilla-La Mancha. RC	2013	Cuenca, Ciudad Real, Horcajo de Santiago, Iniesta, Mota del Cuervo, Quintanar del Rey. San Clemente, Villanueva de la Jara, Tarancón, Motilla del Palancar, Daimiel, Manzanares, Puertollano, Miguelturra, Campo de Criptana, Villarrubia de los ojos, Bolaños de Calatrava, Herencia, Tomelloso (Castilla-La Mancha)	735
ESCA	Generalitat Cataluña. GB	2015–2016	Alt Pirineu I Aran, Barcelona, Camp de Tarragona, Girona, Lleida, Terre de l´Ebre(Cataluña)	419
PREFIT	University of Granada. RC	2014	Almería, Cádiz, Castellón de la Plana, Cuenca (Castilla La Mancha), Granada (Andalucía), Las Palmas (Islas Canarias), Madrid (Madrid), Mallorca, Vitoria (País Vasco), Zaragoza (Aragón), Castellón, (Valencia), Cuenca	2867
Cuenca	University of Castilla-La Mancha. RC	2015	Albacete, Ciudad Real, Cuenca, Guadalajara, Tomelloso, Almansa, Ontur, Villarrobledo, Alcoba, Argamasilla de Calatrava, Azuqueca de Henares, Cabezarrubias del puerto, Cañaveras, El Real de San Vicente, El Robledo, Fuensalida, Gálvez, La Solana, Landete, Las perdices, Los Yebenes, Madridejos, Mestanza, Miguel Esteban, Talavera de la Reina, Totanes, Villares del Saz, Viso del Marqués(Castilla-La Mancha)	413
Barcelona	Autonomous University of Barcelona. RC	2017	Terrassa (Cataluña)	353
N = 5		2013–2017	66 localities	4787

When the name of the study was inexistent, the name of the city where the research centre /government institution that conducted that study was included.
